# Impact of peripheral muscle strength on extubation success after cardiac surgery

**DOI:** 10.31744/einstein_journal/2026AO1455

**Published:** 2026-03-13

**Authors:** Júlio Adriano Leal de Bittencourt Carvalho, Maria Beatriz Sampaio Santana, André Luiz Lisboa Cordeiro

**Affiliations:** 1 Centro Universitário Nobre Feira de Santana BA Brazil Centro Universitário Nobre, Feira de Santana, BA, Brazil.

**Keywords:** Weaning, Thoracic surgery, Airway extubation

## Abstract

**Objective:**

To evaluate the effects of peripheral muscle strength on extubation success after cardiac surgery.

**Methods:**

In this prospective cohort study, peripheral muscle strength and Rapid Shallow Breathing Index were assessed at 30 and 10 minutes during a spontaneous breathing trial. Patients were extubated and monitored for 48 hours to verify the success or failure of extubation, and preoperative variables were compared.

**Results:**

A total of 66 patients were evaluated, 55 (83%) of whom were classified as successful and 11 (17%) as weaning failures. The Medical Research Council score 30 minutes before the spontaneous breathing trial demonstrated a cut-off value of 44, with a sensitivity of 77%, specificity of 84%, area under the curve of 0.864, and 95% confidence interval (95%CI) 0.69-1.00. The Medical Research Council score 10 minutes before the spontaneous breathing trial showed a cut-off value of 49, with a sensitivity of 55%, specificity of 80%, area under the curve of 0.845, and 95%CI= 0.77-1.00. The Rapid Shallow Breathing Index assessed 10 minutes before the spontaneous breathing trial had a cut-off value of 45, with a sensitivity of 30%, specificity of 70%, area under the curve of 0.476, and 95%CI= 0.22-0.71.

**Conclusion:**

Peripheral muscle strength may be a useful predictor of extubation success in patients undergoing cardiac surgery.

## In Brief

Peripheral muscle strength may be a reliable predictor of extubation success in patients undergoing cardiac surgery, outperforming the Rapid Shallow Breathing Index.

## Highlights

■ Peripheral muscle strength outperformed the Rapid Shallow Breathing Index in predicting extubation success.
■ Extubation was successful in 83% patients within a 48-hour monitoring period.
■ Peripheral muscle strength showed strong predictive accuracy.
■ Rapid Shallow Breathing Index demonstrated limited value as a predictor of extubation success.

## Graphical abstract

**Figure:**
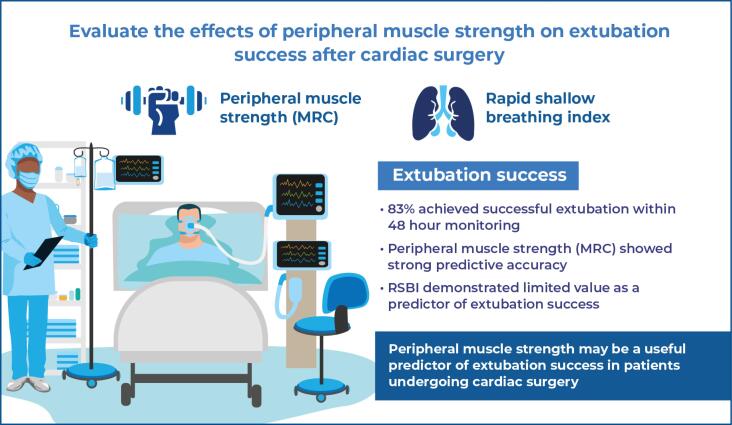

## INTRODUCTION

Patients admitted to the Intensive Care Unit (ICU) frequently develop peripheral and respiratory muscle weakness. This condition is associated with delayed weaning and/or extubation failure, mainly due to respiratory muscle dysfunction and/or inadequate coughing.^(1)^ Although an experienced clinician can predict the likely outcome of an attempt to wean a patient from mechanical ventilation (MV), it is desirable to have predictive indices that can be easily measured and widely applied.^(2)^

Several tests have been devised to predict extubation success after cardiac surgery, including the Medical Research Council (MRC) scale, which has been used to demonstrate the negative impact of muscle weakness on weaning from MV.^(1)^

Determining the ideal time to discontinue MV is critical, because if extubation fails, reintubation is necessary, increasing the risk of mortality.^(3)^ Extubation is a complex process that requires a balance between the overall respiratory load and the ability to overcome this load to wean a patient off from invasive MV.^(4)^

Extubation failure and reintubation occur in 2-25% of mechanically ventilated patients in the ICU.^(5)^ The rate may vary depending on the type of patient and weaning protocol used. Extubation failure and reintubation are associated with increased MV time, mortality, need for tracheostomy, and hospital costs.^(5,6)^

Parameters such as the ability to stick out the tongue, follow specific commands, and having a gag reflex have been investigated as reliable tools for assessing the level of consciousness and ability to protect the airway.^(6)^ Accordingly, several tests have been proposed to predict extubation success after cardiac surgery. Previous studies have used the MRC scale to demonstrate the negative impact of muscle weakness on weaning from MV.^(1)^ However, no study has examined whether peripheral muscle strength can be used as a predictor of weaning success in patients undergoing cardiac surgery.

## OBJECTIVE

To evaluate the impact of peripheral muscle strength on extubation success after cardiac surgery.

## METHODS

### Design

This prospective cohort study included patients admitted to the ICU of the *Instituto Nobre de Cardiologia in Feira de Santana*, Bahia, between January 2019 and September 2021. This study was approved by the Ethics and Research Committee of the *Faculdade Nobre de Feira de Santana* (CAAE: 39988414.0.0000.5654; #917.294). All participants signed an informed consent form.

### Inclusion and exclusion criteria

We included individuals of both sexes aged >18 years who underwent myocardial revascularization with extracorporeal circulation and median sternotomy. Patients with changes in the level of consciousness, cognitive and physical limitations that compromised the assessment, death, emergency surgery, surgical re-intervention, bleeding from drains or surgical incision, and those with medical contraindications for weaning were excluded.

### Study protocol

The patients’ peripheral muscle strength was assessed preoperatively using the MRC scale. Respiratory muscle strength was assessed using the maximum inspiratory pressure (MIP), maximum expiratory pressure (MEP), and pulmonary function parameters, including the vital capacity (VC) and peak expiratory flow (PEF). Data on clinical and surgical characteristics such as diabetes mellitus, systemic arterial hypertension, dyslipidemia, acute myocardial infarction, and sedentary lifestyle were collected from the patients’ medical records, with the exception of sedentary lifestyle. For assessing the lifestyle, the International Physical Activity Questionnaire (IPAQ) was applied in the long format. This questionnaire consists of 27 questions related to physical activities performed in a normal week, with light, moderate, and vigorous intensity lasting for 10 continuous minutes. Physical activities are divided into four categories, including work, transportation, domestic activities and leisure. Those who did not perform any physical activity for at least 10 minutes continuously during the week were considered to have a sedentary lifestyle.^(7)^

After the evaluation, the patients were sent to the surgical center and transferred to the ICU. The surgical procedure was always performed by the same team and the same protocol was adopted for all patients without interference from the researchers.

Upon arrival at the ICU, patients were started on MV using the following parameters: tidal volume of 6-8ml/kg, PEEP 0.5 cmH2O, and inspired oxygen fraction of 60%. The entire process of ventilatory assistance and general care was carried out in accordance with the routine of the Physiotherapy, Medical and Nursing teams. The presenting condition for weaning and peripheral muscle strength were again assessed. Weaning was initiated with a spontaneous breathing test at a pressure support of 7cmH_2_O, PEEP of 5cmH_2_O, and FiO2 <40%. Weaning was performed while maintain clinical, hemodynamic, and gasometric stability. After the patient was extubated, low-flow supplemental oxygen was initiated, with the aim of maintaining peripheral oxygen saturation (SpO2) of 94-97%.

After extubation, the patients were monitored for 48 h to verify the success or failure of extubation. Weaning was considered successful if the patient did not return to ventilatory support within the 48-hour period.

### Measured variables

The MIP was assessed using an analog manovacuometer from Indumed^®^. Using a face mask, the patient was asked to exhale as much as possible until the residual volume was reached and then slowly inhale as much as possible until the total lung capacity was achieved. This test was performed using the unidirectional valve method, allowing a flow rate through a one-millimeter orifice to exclude the action of the buccinator. The test was repeated thrice, and the highest value achieved was recorded, provided that this value was not the last.^(8)^

The MEP was measured using the same device, and the patient was instructed to take a deep breath until the total lung capacity (TLC) was reached. Then, the mask was placed and the patient was asked to take a deep breath until residual capacity was reached. The test was repeated three times, and the highest value was considered, provided it was not the last.^(8)^

To assess the VC, a face mask connected to the expiratory branch of an analog respirometer (Ferraris - Mark 8 Wright Respirometer, Louisville, CO, USA), and the patient was instructed through all phases of the test. The respirometer was unlocked, reset, and a face mask was placed on the patient's face. The patient took a deep breath until the total pulmonary capacity was reached, and then exhaled slowly and gradually until the residual volume was reached. Subsequently, the spirometer was locked, and the result was recorded. The test was repeated three times, and the highest value was included in the analysis.^(9)^

Peak expiratory flow was assessed using the Mini Wright^®^ Peak Flow. During the assessment, the patient was seated with the head in a neutral position and a nose clip was placed to prevent air from escaping through the nostrils. The patient took a deep breath, up to full lung capacity, followed by forced exhalation with the mouth on the device. After three measurements, the highest value was selected, ensuring that the difference was not greater than 40 liters between the measurements.^(9)^

The MRC scale measures the peripheral muscle strength by evaluating the ability of six muscle groups (shoulder abductors, elbow flexors, wrist extensors, hip flexors, knee extensors, and ankle dorsiflexors) to overcome the load, scoring each muscle group bilaterally from 0 to 5, where zero indicates no contraction and five indicates overcoming the maximum resistance imposed by the examiner. The minimum score for this test is 0 (tetraplegia) and the maximum can reach 60 points (preserved muscle strength). A score below 48 may be suggestive of polyneuromyopathy.^(10)^

### Statistical analysis

The data were analyzed using SPSS version 20.0 software. Data normality was verified using the Shapiro-Wilk test. Data are expressed as means and standard deviations or absolute values and percentages. Comparisons between groups were performed using independent Student's *t*-test. The optimal MRC cut-off value was determined using receiver operating characteristic (ROC) curve analysis. A two-sided p<0.05 was considered statistically significant.

## RESULTS

**Figure 1:**
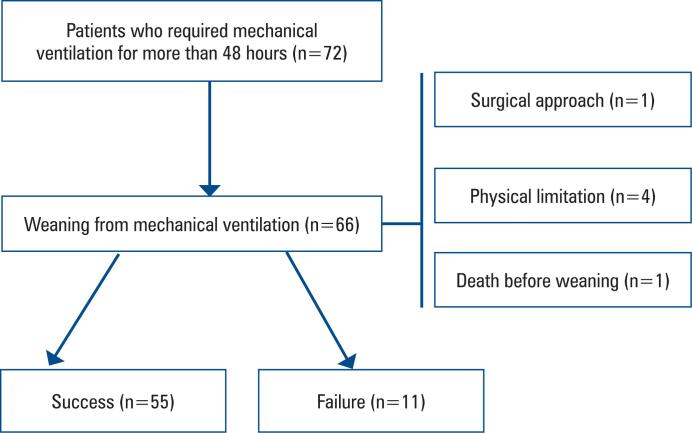
Flowchart showing the inclusion and exclusion criteria

During the study period, 72 patients required MV, six of whom were excluded from the study (Figure 1). Of the remaining 66 patients, 55 (83%) were classified as successful extubation and 11 (17%) as having failed weaning. The majority of the patients were men (38 [58%]) and the overall mean age was 62 ± 5 years. The most prevalent comorbidities were systemic arterial hypertension and sedentary lifestyle, affecting 49 (74%) and 45 (68%) participants, respectively. Other relevant information is presented in table 1.

**Table 1:** Clinical and surgical characteristics of the included patients

Variable		n=66
Sex, n (%)		
	Male	38 (58)
	Female	27 (42)
Age (years)		62±5
Body mass index (kg/m^2^)		26±3
Comorbidities, n (%)		
	Systemic arterial hypertension	49 (74)
	Dyslipidemia	38 (58)
	*Diabetes mellitus*	41 (62)
	Acute myocardial infarction	9 (14)
	Sedentary lifestyle	45 (68)
Surgery time (minutes)		231±74
Extracorporeal circulation time (min)		92±15
Aortic clamping time (minutes)		79±12
Mechanical ventilation time (hours)		6±3
Left ventricular ejection fraction (%)		45±5
Weaning outcome, n (%)		
	Weaning success	55 (83)
	Weaning failure	11 (17)

Table 2 presents a comparison of the weaning success and failure groups based on the patient characteristics. Notably, the MRC score 30 minutes before the spontaneous breathing trial (SBT) was significantly higher in the success group (mean 44 ± 4) compared to the failure group (29 ± 5; p<0.001). Similarly, the MRC score 10 minutes before the SBT was 45 ± 5 in the success group and 34 ± 4 in the failure group (p<0.001).

**Table 2:** Comparison of the patient characteristics, peripheral muscle strength, and rapid shallow breathing index between the weaning success and failure groups

Variable	Total	Success	Failure	p value
	n=66	n=55	n=11	
Age (years)	62 ± 5	58 ± 6	64 ± 5	0.78
Male, n (%)	38 (58)	32 (58)	6 (54)	0.46
BMI (kg/m^2^)	26 ± 3	28 ± 3	25 ± 4	0.32
Hypertension, n (%)	49 (74)	42 (76)	7 (64)	0.37
Diabetes, n (%)	41 (62)	35 (64)	6 (54)	0.62
MRC 30 minutes before SBT	40 ± 3	44 ± 4	29 ± 5	<0.001
MRC 10 minutes before SBT	45 ± 5	48 ± 5	34 ± 4	<0.001
RSBI 10 minutes before SBT	44 ± 9	40 ± 7	46 ± 9	0.56

BMI: body mass index; MRC: Medical Research Council; SBT: spontaneous breathing test; RSBI: rapid shallow breathing index.

**Figure 2:**
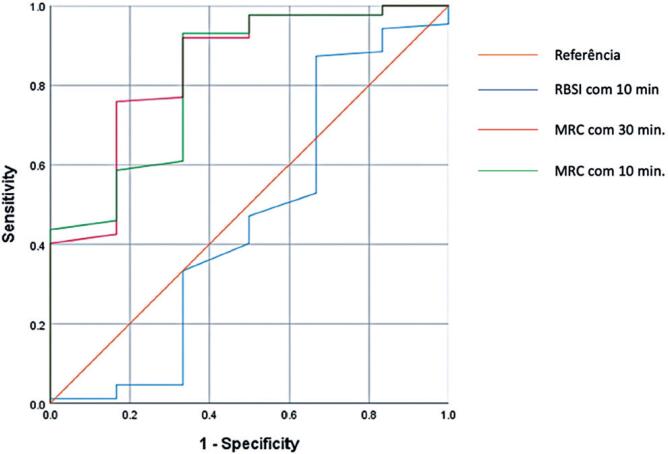
Receiver operating characteristic (ROC) curve

The study identified a cut-off value of 44 for the MRC score 30 minutes before the SBT, which yielded a sensitivity of 77%, specificity of 84%, area under the curve (AUC) of 0.864, and 95% confidence interval (95%CI): 0.69-1.00. For the MRC score 10 minutes before the SBT, a cut-off value of 49 was associated with a sensitivity of 55%, specificity of 80%, AUC of 0.845, and 95%CI= 0.77-1.00. In comparison, the rapid shallow breathing index (RSBI) measured 10 minutes before the SBT had a cut-off value of 45, with a sensitivity of 30%, specificity of 70%, AUC of 0.476, and 95%CI= 0.22-0.71 (Table 3). These data are demonstrated in figure 2.

**Table 3:** Cut-off values for peripheral muscle strength and rapid shallow breathing index in the weaning success and failure groups

Variable	Cut-off value	AUC	95% CI	Sensitivity (%)	Specificity (%)	PPV (%)	NPV (%)	p value
MRC 30 minutes before SBT	44 ± 4	0.864	0.69-1.00	77	84	52	49	0.004
MRC 10 minutes before SBT	49 ± 5	0.845	0.77-1.00	55	80	44	58	0.005
RSBI 10 minutes before SBT	45 ±4	0.476	0.22-0.71	30	70	40	65	0.84

MRC: Medical Research Council; SBT: spontaneous breathing test; RSBI: rapid shallow breathing index; PPV: positive predictive value; NPV: positive negative value.

## DISCUSSION

This study aimed to evaluate the peripheral muscle strength by using a new MV weaning success index. The sensitivity and specificity of the MRC were found to be 77% and 84%, respectively, whereas the RSBI demonstrated sensitivity and specificity of 30% and 70%, respectively. These findings reveal that the MRC is more accurate than the RSBI for assessing the peripheral muscle strength 30 and 10 minutes before the SBT.

A study of neurocritical patients by Kutchak et al. reported that the RSBI was one of the most commonly used parameters in the ICU, but it was only partially capable of predicting the risk of extubation failure. Regardless of the restitution of the primary pathology that leads to intubation in neurological patients, the motor and cognitive sequelae lead to a decreased capacity to protect the airways.^(11)^

Previous studies have proposed that muscle weakness associated with increased respiratory work can result in a rapid and shallow breathing pattern. However, Saiphoklang et al. found a correlation between the handgrip strength and RSBI in cases of pneumonia, pulmonary edema, and bronchospasm, and demonstrated the non-specificity of the RSBI in distinguishing between patients who were successfully extubated and those who failed.^(12)^

Faadaii et al. examined the usefulness of RSBI as a predictor of weaning in the ICU, and found that it had low specificity compared to variables such as concomitant diseases and length of stay in the ICU.^(13)^ In an investigation into the correlation between decreased skeletal muscle mass and extubation failure, Woo et al. inferred that the RSBI is not capable of predicting extubation success, and has sensitivity and specificity of 0.74 and 0.00, respectively.^(14)^

Consequently, the MRC scale is more practical and viable for predicting extubation success. However, it does not eliminate or replace other assessment methods. Lima et al. evaluated the influence of peripheral muscle strength and other indicators on tracheostomy decannulation success, and found that the MRC value was greater than or equal to 26 in the successful decannulation group, with a sensitivity of 94.4% and specificity of 50%. This study showed that peripheral muscle strength evaluated on the day of decannulation may affect the tracheostomy decannulation success rate.^(15)^

The MRC scale has been frequently used as a practical means of recognizing muscle weakness acquired in the ICU. In a study by Santos et al. in critically ill oncology patients, it was shown that patients whose MRC score was lower at extubation remained on MV for a longer period of time. This finding supports an association between peripheral muscle weakness and weaning failure.^(16)^

Regarding the functional prognosis of patients after hospitalization in the ICU, Ferreira et al. showed that severe clinical conditions at admission can contribute to a subsequent decline in the functional performance. Thus, the MRC scale may serve as an indicator for the early implementation of rehabilitation.^(17)^

This study has some limitations. First, other predictive indices of weaning and extubation were not evaluated in this study. Second, a pain assessment scale was not used, which may also influence weaning. Third, the limited sample size may have influenced the study results.

Additionally, it is important to acknowledge potential confounding variables, such as age, comorbidities, surgical time, and duration of MV, which were not adjusted for in the analysis and may have significantly affected both the MRC scores and extubation outcomes. Finally, the lack of multivariate analysis limits the ability to confirm the independent effects of peripheral muscle strength on weaning success.

## CONCLUSION

Therefore, peripheral muscle strength may be a useful predictor of extubation success in patients undergoing cardiac surgery. However, further studies with larger sample sizes and multivariate analyses are required to confirm this association.

## Data Availability

The underlying content is contained within the manuscript.
